# Worry, Risk Perception, and Controllability Predict Intentions Toward COVID-19 Preventive Behaviors

**DOI:** 10.3389/fpsyg.2020.582720

**Published:** 2020-11-19

**Authors:** Agata Sobkow, Tomasz Zaleskiewicz, Dafina Petrova, Rocio Garcia-Retamero, Jakub Traczyk

**Affiliations:** ^1^Faculty of Psychology in Wrocław, Center for Research on Improving Decision Making (CRIDM), SWPS University of Social Sciences and Humanities, Wrocław, Poland; ^2^Faculty of Psychology in Wrocław, Center for Research in Economic Behavior, SWPS University of Social Sciences and Humanities, Wrocław, Poland; ^3^Escuela Andaluza de Salud Pública, Granada, Spain; ^4^Instituto de Investigación Biosanitaria ibs.GRANADA, Granada, Spain; ^5^CIBER of Epidemiology and Public Health (CIBERESP), Madrid, Spain; ^6^Department of Experimental Psychology, University of Granada, Granada, Spain

**Keywords:** COVID-19, risk perception, preventive behaviors, worry, controllability, numeracy, mental imagery, affect

## Abstract

The ongoing pandemic of COVID-19 has already had serious worldwide health, socio-economic, political, and educational consequences. In the present study, we investigated what factors can motivate young adults to comply with the recommended preventive measures against coronavirus infection. Even though young people are less likely to suffer severe medical consequences from the virus, they can still transmit it to more vulnerable individuals. Surprisingly, we found no significant effects of previously successful experimental manipulations (e.g., enhancing self-efficacy, and visual aids) that aimed to improve risk understanding and impact COVID-19 related behavioral intentions. Instead, intentions toward preventive behaviors were predicted by self-reported worry, perceived controllability of the pandemic, and risk perception. Interestingly, worry about health, and worry about restricting personal freedom predicted behavioral intentions in diverging directions. In particular, participants who were worried about health, were more willing to obey strict hygiene and social distancing restrictions. In contrast, participants who were worried about personal restrictions, were less ready to adopt these preventive actions.

## Introduction

The ongoing pandemic of coronavirus disease 2019 (COVID-19) caused by the severe acute respiratory syndrome coronavirus 2 (SARS-CoV-2) has already had serious worldwide health, socio-economic, political, and educational consequences (European Commission, 2020; Van Bavel et al., 2020; World Health Organization, 2020). Even though governments around the world adopted different response strategies to tackle the pandemic, at some stage most countries either enforced or encouraged policies targeting preventive behaviors such as social distancing (Petherick et al., 2020). These included, among others, school and restaurant closures, working from home, or not going out unless absolutely necessary, all aimed at avoiding physical contact and transmission of the virus.

Data show that the elderly and those with chronic diseases are the groups most vulnerable to the virus (Zheng et al., 2020), whereas young people in good health generally tend not to suffer severe consequences if infected. However, young people’s collaboration in the efforts to stop the virus from spreading is essential because they can be transmission vectors. Initial data from Italy collected at the height of the pandemic indicate that, albeit compliance with preventive behaviors was high overall, younger adults (<40) reported lower compliance (Barari et al., 2020; a non-peer-reviewed preprint). This was especially the case for keeping physical distance from others and washing hands more frequently. Similar results—lower self-reported compliance with COVID-19 preventive behavior among younger adults—were also observed in the United Kingdom (Fancourt et al., 2020). These results suggest that age-targeted messages may be needed to increase compliance (Utych and Fowler, 2020) and that research identifying factors that can help increase compliance with preventive behaviors among younger people is needed.

### Factors Influencing Risk Perception and Behavioral Intentions Toward Preventive Behaviors

People’s behavior under threat may depend on how they perceive risk (e.g., Brewer et al., 2007). Following models developed earlier by Slovic (1987, 2016), we define risk perception in terms of the subjective, intuitive judgment that people make about risk with regard to its size and multidimensional nature. A bulk of research from the field of Judgment and Decision Making (see for a review, Loewenstein et al., 2001; Slovic, 2013; Keren and Wu, 2015; Lerner et al., 2015; Zaleskiewicz and Traczyk, 2020) demonstrated that various factors (e.g., cognitive or emotional) might influence the perception of risk, which means that risk perception is constructed as a general view people have about the severity of danger and is determined by affect, prior experience, and simple evaluations of threats/benefits, among others. Additionally, risk perception is a crucial predictor of preventive behaviors. For example, Bruine De Bruin and Bennett (2020) showed that individuals who perceived risk related to COVID-19 as higher (i.e., higher chances for SARS-CoV-2 infection and infection fatality) declared that they were more likely to implement protective behaviors. In the present research, we aimed to explore potential mechanisms that may underlie risk perception and behavioral intentions toward COVID-19 in young adults. We based our predictions on the risk-as-feelings hypothesis (Loewenstein et al., 2001) as the main theoretical model describing the role of various factors in risk perception and behavior under risk. Within this model, risk-related behavior results from a dynamic interplay between cognitive evaluations and feelings that arise from anticipated outcomes, subjective probabilities as well as other factors such as vividness of a threat (e.g., vividness of mental images of risk). We review these risk-related factors in the following sections.

#### Affect

Different decision-making models have indicated that one of the factors that has the capacity to regulate risk perception is affect (Loewenstein et al., 2001; Bechara and Damasio, 2005; Pfister and Böhm, 2008; Mohr et al., 2010; Lempert and Phelps, 2013; Lerner et al., 2015; Parrott, 2017; Zaleskiewicz and Traczyk, 2020). Lerner et al. (2015) even proposed that “emotions are, for better or worse, the dominant driver of most meaningful decisions in life” (p. 801). The popular psychological approach to the understanding of risk perception—psychometric paradigm (Fischhoff et al., 1978; Slovic et al., 1982; Slovic, 1987; Weber, 2017; Visschers and Siegrist, 2018)—suggests that perceived risk can be represented by two dimensions which are named “unknown risk” and “dread risk,” with the latter being associated with emotions. The more fear people experience when being exposed to risk, the more they tend to judge risk as higher (Slovic, 1987; Marris et al., 1997; Siegrist et al., 2005). In an independent stream of research, Lerner and Keltner (2000, 2001) found that both dispositional and incidentally evoked fear was related to higher risk estimations, which further supports the idea that risk perception may be driven by affective influences. Moreover, the strength of worry has been shown to be positively related to risk estimation for different types of risk (e.g., health risk, environmental risk, and financial risk; Holtgrave and Weber, 1993; Koonce et al., 2005; Weber and Stern, 2011) as well as preventive behaviors, such as buying insurance against natural disasters (Zaleskiewicz et al., 2002; Sobkow et al., 2017).

Having all these effects in mind, we expected that both people’s perceptions of threats related to the COVID-19 pandemic and their protective actions would be predicted by the affective factor of worry. More precisely, we hypothesized that when people report more worry when thinking about the pandemic, they tend to provide higher risk estimations and declare a stronger need to undertake protective behaviors. However, negative emotions such as fear or worry could also have negative consequences in case of dealing with a disaster. For example, previous research indicated that people experiencing fear and uncertainty (such as COVID-19 outbreak) tend to buy more things than usual (i.e., panic buying, Arafat et al., 2020; Lins and Aquino, 2020; Sim et al., 2020). Moreover, based on the recent research by Peters et al. (unpublished; see also Peters, 2020), which showed that obsessing over daily coronavirus statistics might be counterproductive, we hypothesized that statistics stalking would be positively related to worry and panic buying.

#### Mental Imagery

Theoretical models (Lang, 1979; Loewenstein et al., 2001; Ji et al., 2016) accompanied by empirical evidence (Peters and Slovic, 1996; Holmes and Mathews, 2005, 2010; Leiserowitz, 2005) have pointed at mental imagery as one of the sources of emotions in judgment and decision making. Recent research had documented that when people produced more vivid, negative mental images associated with risk, they tended to estimate risk as higher and that the relation between negative mental imagery and risk perception was mediated by feelings of stress (Traczyk et al., 2015; Sobkow et al., 2016). However, less attention was paid to the potential role of positive mental imagery in the risk-appraisal process. Risky or uncertain situations may be seen not only as a source of threat, but also as a chance to gain some benefits (Weber et al., 2002); therefore, they have the capacity to reinforce the production of not only negative but also positive mental images. For example, in the context of the pandemic, people can imagine themselves as suffering severe health consequences (negative mental imagery) but also as strengthening relations within their families because of staying at home (positive mental imagery). It is suggested (Van Bavel et al., 2020) that using a positive frame may relieve negative emotions and educate the public in case of the COVID-19 pandemic. However, potentially, the easiness with which people create positive imagery can be seen as a factor that hampers their need to undertake protective actions because it promotes more optimistic views of the future and endorses approach motivation (Escalas and Luce, 2003; Armitage and Reidy, 2008). Even if positive imagery of living under the pandemic crisis may have some beneficial side effects for undertaking protective behaviors (i.e., people should be more willing to stay at home if they create positive mental images of spending more time with their relatives), we do believe that in most cases it would increase unreasonable behaviors, as a result of strengthening highly (sometimes unrealistically) optimistic perception of the situation. Importantly, recent research (Kulesza et al., 2020) demonstrated that the effect of unrealistic optimism regarding chances of being infected with SARS-CoV-2 was especially pronounced in young adults (students) in comparison to healthcare professionals.

In the present project, we encouraged one randomly selected group of participants to create positive mental images related to the COVID-19 pandemic to investigate their impact on risk perception. We hypothesized that imagining positive consequences of the pandemic would decrease negative affect, but also that it would be linked to lower risk estimations and intentions toward preventive behaviors (in comparison to a control condition). We would like to note that our participants were not asked to simply prepare a list of potential consequences of being exposed to a threat (i.e., listing and assessing consequences is typically used in the decision-making research), but to create a vivid visual (and positive, in this case) representation of what may happen to them.

#### Controllability and Self-Efficacy

Cognitive evaluations and risk-related feelings may also be driven by characteristics of a specific threat, such as its controllability (Loewenstein et al., 2001). Slovic (1987) argued that a perceived lack of control (along with being catastrophic or having fatal consequences) is highly correlated with a “dread risk”—an emotional dimension of risk perception. Nevertheless, other research (e.g., Fischhoff et al., 1978; Siegrist et al., 2005) suggested that uncontrollability is also related to a cognitive dimension such as “unknown risk”/“unobservable hazards” (along with involuntariness or newness). Controllability could be considered not only as a factor shaping risk perception, but also as a tool that might be used to design effective interventions aimed to influence preventive behaviors. In particular, Bandura (1982, p. 126) argued that controllability and predictability “are conducive to the enhancement of self-percepts of efficacy” and high self-efficacy—“judgments of how well one can execute courses of action required to deal with prospective situations” (Bandura, 1982, p. 122)—is beneficial for performance in various domains such as health (Bandura, 1982, 1990; Luszczynska et al., 2009; Gwaltney et al., 2013), business (Stajkovic and Luthans, 1998; Miao et al., 2017), and sport (Moritz et al., 2000). Moreover, fear appeals (persuasive messages that arouse fear) are found to be effective (led to behavioral changes) only when individuals feel capable of dealing with the threat (Witte and Allen, 2000). That is, when people experience intense fear but feel helpless, such appeals could provoke defensive responses.

In the present project, besides measuring subjective controllability of the pandemic and perceived effectiveness of social distancing, we introduced an experimental manipulation of state self-efficacy. One randomly selected group of participants was encouraged to describe what measures they could take to protect themselves and their families from the negative consequences related to the COVID-19 pandemic. We hypothesized that thinking about what people could do to protect themselves or their families would reduce negative emotions, increase controllability, and increase intentions toward preventive behaviors (in comparison to a control condition).

#### Numeracy

According to the risk-as-feelings hypothesis (Loewenstein et al., 2001), cognitive evaluations and risk-related feelings might also be influenced by subjective probabilities associated with a threat. However, many people, including those well-educated, experience difficulties when faced with numerical information (Lipkus et al., 2001) such as SARS-CoV-2 cases or infection fatality. Those who properly understand statistical and probability information and use it appropriately in everyday contexts—individuals with high statistical numeracy—are usually more risk literate (Cokely et al., 2018). They better understand and evaluate risks, what can result in generally better decisions in various domains, from health to finance (Reyna et al., 2009; Cokely et al., 2018; Garcia-Retamero et al., 2019; Sobkow et al., 2020a). Several psychological mechanisms may underlie better performance of people with high numeracy. These mechanisms are not limited to performing mathematical operations; such individuals often use elaborate heuristics search (Cokely and Kelley, 2009), deliberate more on decision problems, are more consistent in processing probabilities (Traczyk et al., 2020), and have a more accurate evaluation of their judgments (Ghazal et al., 2014), as well as search for more information (Ashby, 2017; Traczyk et al., 2018a). Finally, they adaptively change the strategy based on the structure of decision problem (Traczyk et al., 2018b) and use affect as an important clue in the decision-making process, when it is related to decision problem (Peters et al., 2006; Petrova et al., 2014), but not when it is incidental (Traczyk and Fulawka, 2016).

In addition, recent research demonstrated that numeracy is not a unitary construct (Peters and Bjälkebring, 2015; Sobkow et al., 2020b). Different components of numeracy such as subjective numeracy (preference for numerical format and confidence with numbers) or approximate numeracy (an ability to perceive and manipulate numerosities and to map symbolic numbers to magnitudes) might predict distinct decision outcomes from statistical numeracy. We hypothesized that different types of numeracy would be related to COVID-19 forecasts, risk perception, and intentions toward preventive behaviors.

#### Visual Aids

One of the methods that could help people (especially those with low numeracy) better comprehend risk is based on a presentation of numerical information in the form of simple graphical representations of numerical expressions—visual aids. These visual aids might have a form of icon arrays, bar and line charts, and others (Ancker et al., 2006; Spiegelhalter et al., 2011; Hildon et al., 2012). Visual aids have long been known to confer benefits when communicating risk information about health (Zikmund-Fisher et al., 2008; Gaissmaier et al., 2012; Garcia-Retamero et al., 2020), promoting consideration of beneficial treatments despite side effects (Waters et al., 2007), informing patients’ decisions about effective medical interventions and their influence on the quality of life (Brundage et al., 2005), and increasing the probability of health-promoting behaviors (Garcia-Retamero and Cokely, 2011). Importantly, visual aids were also found to be effective in the context of the Ebola epidemic in 2014 in the United States: individuals who received visual aids showing the risk of getting infected with Ebola and the risk of dying once infected, reported more accurate risk comprehension, which also translated into reduced fear and healthier behavioral intentions (Petrova, 2016).

However, not all visual aids are equally effective. Visual aids tend to provide an efficient means of risk communication when they are transparent (Garcia-Retamero and Cokely, 2013, 2017)—that is, when they promote representative (or unbiased) risk understanding and evaluation. Generally, this transparency means that the elements of the visual aid are well defined, and they accurately and clearly represent the essential risk information by making part-to-whole relationships in the data visually available and comparable (Garcia-Retamero and Cokely, 2017).

In the present research, we designed two visual aids: one representing the cumulative number of SARS-CoV-2 cases in a single country (Poland) and another one showing statistics from different countries (including Poland). We hypothesized that both types of visual aids would improve risk understanding (in comparison to a control condition in which participants received no visual aid)—that is, participants receiving a visual aid would provide better estimates and forecasts of SARS-CoV-2 cases in Poland. Moreover, a visual aid showing statistics from different countries would improve estimates and forecasts of SARS-CoV-2 cases compared to the visual aid condition reporting only data in Poland.

### Aims of the Study

Informed by the risk-as-feelings framework (Loewenstein et al., 2001), the aim of the current study was to test what psychological factors may predict people’s intentions toward COVID-19 preventive behaviors and other outbreak responses. We explored the role of individual differences (i.e., statistical, approximate, and subjective numeracy) as well as emotional and cognitive factors (e.g., controllability and risk perception, worry elicited by COVID-19 pandemic: related to health, restrictions, and financial consequences). We also tested whether different interventions (i.e., boosting self-efficacy, evoking positive mental images of pandemic consequences, introducing visual aids related to one country and in comparison to other countries) could influence the willingness to take preventive measures against SARS-CoV-2 for a longer period of time.

## Materials and Methods

### Participants

Two hundred and fifty-three students from Poland completed an online questionnaire (*M*_age_ = 29.2, *SD*_age_ = 9.3, *Mdn* = 26.0; 221 females; 65 participants had children; 61 participants lived with older or chronically ill persons; 162 participants were employed, and 111 of them could work online). Participants took part in the study in exchange for credit points (only data from participants who completed the whole procedure were taken into account in the analyses). Participation in the study was voluntary, and participants gave informed consent before the study. The study protocol was approved by the departmental Ethical Committee.

### Measures

#### Individual Differences

Participants completed measures of individual differences in multiple numeric competencies: statistical numeracy, subjective numeracy, and approximate numeracy that were found to be important predictors of decision outcomes (Peters and Bjälkebring, 2015; Sobkow et al., 2020a, b). This measurement was administered about 14 days before the main study.

##### Statistical Numeracy

Statistical numeracy was measured by the 4-item Berlin Numeracy Test (BNT; Cokely et al., 2012). The items involved tasks measuring understanding of statistics and probability (e.g., “Imagine we are throwing a five-sided die 50 times. On average, out of these 50 throws how many times would this five-sided die show an odd number?”). Possible scores on the test ranged from 0 to 4 points, with higher scores indicating higher statistical numeracy (McDonald’s ω = 0.59).

##### Subjective Numeracy

Subjective numeracy was measured by the 8-item subjective numeracy scale (McDonald’s ω = 0.87; Fagerlin et al., 2007). Participants answered each question using a 6-point scale to assess their perceived numerical abilities (e.g., “How good are you at working with percentages?”) and preference for numerical information (e.g., “How often do you find numerical information to be useful?”).

##### Approximate Numeracy

We used a symbolic-number mapping task adopted from previous research (Opfer and Siegler, 2007; Sobkow et al., 2019) to measure approximate numeracy (McDonald’s ω = 0.92). In this task, participants were asked to place a target value on a number line anchored from 0 to 1,000 using a movable slider. We used 22 numbers (i.e., 2, 5, 18, 34, 56, 78, 100, 122, 147, 150, 163, 179, 246, 366, 486, 606, 722, 725, 738, 754, 818, and 938) following those proposed by Opfer and Siegler (2007). Each number was shown in a separate trial presented in random order. At the beginning of each trial, the slider was placed on the left-hand end of the number line (the location of 0). The target number was presented above it. For each participant and each trial, we calculated the absolute deviance from the target number (e.g., if the target number was 16 and a participant placed the slider on 18, the deviance score was 2). Then, we applied a logarithmic transformation to these scores (because of a right-skewed distribution), averaged them across all 22 trials. The measure was recoded in a way that higher scores indicated higher approximate numeracy.

#### Interventions

Participants were randomly assigned to one of five experimental conditions: (1) the control condition, (2) the enhance self-efficacy condition, (3) the positive mental images related to COVID-19 pandemic condition, (4) the visual aid condition receiving a visual aid showing the cumulative number of SARS-CoV-2 cases in Poland, and (5) the visual aid condition receiving a visual aid showing the cumulative number of SARS-CoV-2 cases in Poland in comparison to other countries (i.e., Spain, South Korea, Germany, Norway, and Japan).

We conducted sensitivity analysis with G^∗^Power (Faul et al., 2009). It showed that for a linear regression model, assuming alpha 0.05 and power 0.80, 17 total predictors, and 4 tested predictors (i.e., dummy variables representing the interventions), with the obtained sample size, the study could detect a small effect size of about *R*^2^ = 0.045.

##### Self-Efficacy Condition

In this condition, participants were asked to describe what measures they could take to protect themselves and their families from the negative consequences related to COVID-19 pandemic. They were prompted to describe at least three measures.

##### Positive Mental Imagery Condition

In this condition, participants were asked to imagine and describe potential positive consequences of the COVID-19 pandemic (e.g., there will be a reduced number of flu cases, because of more frequent hand washing; people will be more willing to help each other, and their social attitudes will positively change). They were prompted to describe at least three positive consequences.

##### Visual Aid 1 (Poland) Condition

**FIGURE 1:**
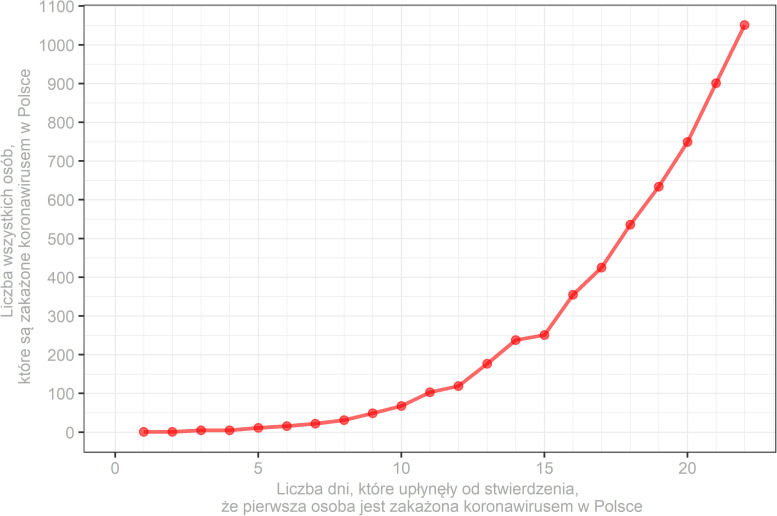
Sample visual aid showing the cumulative number of SARS-CoV-2 cases in Poland (*y*-axis) in consecutive days since the first patient has received a positive test (*x*-axis). Descriptions on the figure are in Polish as they were presented to participants.

In this condition, participants were asked to investigate a graph presenting the cumulative number of SARS-CoV-2 cases in Poland (Figure 1) since the first patient has received a positive test. The data on the graph was updated each day of the study based on the Johns Hopkins University repository (see text footnote 1).

##### Visual Aid 2 (Several Countries) Condition

**FIGURE 2:**
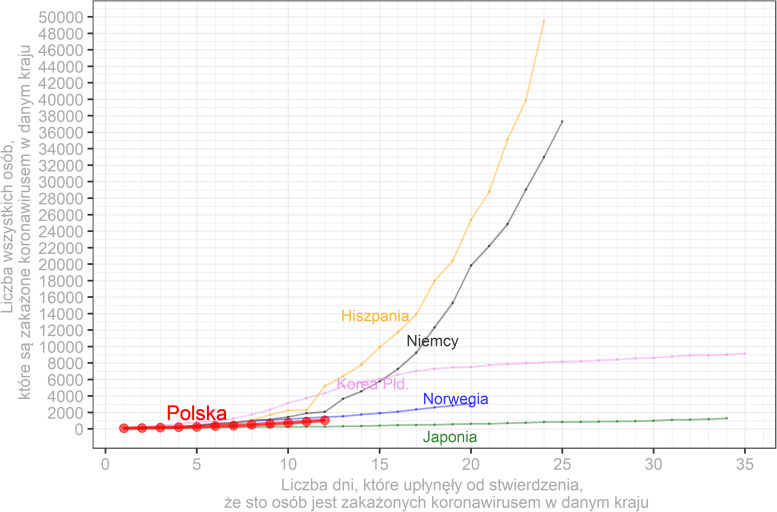
Sample visual aid showing the cumulative number of SARS-CoV-2 cases (*y*-axis) in Poland (red line) in comparison to other countries (Spain—yellow line, Germany—black line, South Korea—pink line, Norway—blue line, Japan—green line) in consecutive days since the 100th patient with a positive test in each country (*x*-axis). Descriptions on the figure are in Polish as they were presented to participants.

In this condition, participants were asked to investigate a graph showing the cumulative number of SARS-CoV-2 cases in Poland in comparison to other countries (i.e., Spain, South Korea, Germany, Norway, Japan) since the 100th case (Figure 2). The data on the graph was also updated each day of the study based on the Johns Hopkins University repository^1^. Moreover, participants received information that countries could differ in terms of the time when protective measures were implemented (e.g., closing public facilities), the number of tests, and the behavior of people (e.g., related to obeying social distancing and hygiene recommendations). Such differences could influence the development of pandemic in a particular country.

#### Psychological Responses to COVID-19

Participants completed several measures of psychological responses to the COVID-19 pandemic, covering a broad spectrum of human functioning: cognitive, emotional, motivational, and behavioral.

##### Intentions Toward Preventive Behaviors

Participants were asked to indicate to what extent they would be willing to take various preventive measures in a longer period of time (e.g., 3 months) using a 7-point scale (1—not at all willing to do it, 7—very willing to do it). The scale contained 21 items covering various measures such as “avoid going to bars or restaurants,” “avoid entering crowded public spaces (e.g., somewhere where there is a queue of people),” “frequently wash hands thoroughly (with soap for at least 30 seconds),” or “disinfect handles, smartphones, etc.” (McDonald’s ω = 0.90).

##### Emotional Responses to COVID-19

Participants were asked to indicate how they felt while thinking about COVID-19 using a 9-point scale (1—not at all, 9—very much) and a list of six adjectives: assured, hopeful, relieved, anxious, afraid, and worried (Garcia-Retamero and Cokely, 2011; Petrova et al., 2015; Petrova, 2016). These questions were combined into a single index with higher scores indicating more positive emotions (McDonald’s ω = 0.85).

##### Sources of Worry About COVID-19 Pandemic

Participants were asked to indicate to what extent they were worried about twenty issues regarding the COVID-19 pandemic using a 7-point scale (1—not at all, 7—very much). Results of the principal component analysis with varimax rotation indicated that there were three components related to different sources of worry about the COVID-19 pandemic. The first component (i.e., worry about health) consisted of 10 items (McDonald’s ω = 0.90) and captured feelings of worry driven by possible health problems related to COVID-19 (e.g., “being hospitalized,” “being sick”). The second component (i.e., worry about restrictions) consisted of six items (McDonald’s ω = 0.77) and described feelings of worry related to perceived social restrictions during COVID-19 (e.g., “being unable to travel,” “being unable to meet friends”). The third component consisted of four items (McDonald’s ω = 0.74) and was related to personal and macroeconomic financial consequences of COVID-19 (e.g., “being unable to work” and “being worried about the rise in unemployment rates”).

##### Panic Buying

Participants were asked to estimate how many items (such as toilet paper, bottles of water, bags of pasta) they have stored at home. Moreover, they were asked to answer how much cash they have stored at home (1–less than usual, 5–more than usual). Because of different response scales, answers for each item were z-scored and then averaged into a single index (McDonald’s ω = 0.56).

##### Statistics Stalking

Participants indicated how often they search for new statistics about COVID-19 pandemic (1—never, 2—once a week, 3—few times a week, 4—once a day, 5—few times a day). This measure was inspired by recent research by Peters et al. (unpublished; see also Peters, 2020) that suggested obsessing over daily coronavirus statistics might be counterproductive.

##### Controllability

Participants completed four questions related to the perception of controllability of the pandemic threat (e.g., “I think that strict compliance with hygiene and social distancing rules makes sense,” “I feel that I can influence whether the members of my family or I get COVID-19,” “People have no influence on the course of the epidemic” [reversed]) using a 7-point scale (1—completely disagree, 7—completely agree). However, after careful inspection of responses, we decided to drop one question (“The epidemic is unpredictable. It is not known how long it will take and how many deaths there will be”). This item was rather related to the threat being perceived as unpredictable (but not necessarily uncontrollable) and it was not related to other items in the scale (dropping this item did not change the general pattern of results obtained in this study). The remaining three questions were combined into a single index (McDonald’s ω = 0.51).

##### Risk Perception of COVID-19

Participants answered five questions related to the perception of risk associated with the COVID-19 outbreak (e.g., “How do you estimate chances that a virus will negatively influence you or your family health?,” “Is this virus a real threat?”) using 5-point scales. These questions were combined into a single index (McDonald’s ω = 0.81). We hypothesized that risk perception would be negatively related to emotional responses to the COVID-19 pandemic and to controllability.

##### COVID-19 Pandemic Forecasts

We asked participants to estimate the current number of SARS-CoV-2 cases in Poland. We specified that we were interested in estimates of the total number of people in Poland who were officially tested and got a positive SARS-CoV-2 result from the first case to the day when the study was taken. Next, each participant was asked to estimate how many people in Poland were going to be test positive with SARS-CoV-2, 1, 2, 3, and four weeks from the day the participant completed the online study. We highlighted that participants had to estimate a cumulative number of cases (i.e., the number of all SARS-CoV-2 cases in Poland that will be announced by the Ministry of Health).

To measure the accuracy of individual estimates of SARS-CoV-2 cases, we calculated to what extent each participant’s estimates deviated from the actual number of cases in Poland. First, we modeled the dynamics of SARS-CoV-2 (i.e., the baseline model) in the period from the beginning of the current study (i.e., March 26^th^, 2020) to the last estimate, 4 weeks after data collection (i.e., April 25^th^, 2020). An increase in SARS-CoV-2 cases was the best described by a linear model (y = 726.060 + 348.267 ^∗^ day; *R*^2^ = 0.997, *p* < 0.001). Second, we fitted individual linear models predicting SARS-CoV-2 cases using participants’ estimates. These models were fitted separately for each participant. Next, to get a measure of the accuracy of individual forecasts for each participant, we calculated the sum of squares of the deviations of every individual model from the baseline model (i.e., the actual number of cases in Poland). This resulted in a measure of dispersion between the baseline and individual models, with higher values indicating higher deviations of individual models from the baseline model. Finally, the measure was log-transformed because of its right-skewed distribution.

##### Perceived Effectiveness of Social Distancing

Apart from providing estimates of SARS-CoV-2 cases, participants were also instructed to estimate how many people were going to test positive for SARS-CoV-2 in Poland, 1, 2, 3, and 4 weeks after that point in time, if the majority of people in Poland followed (i.e., optimistic condition) or did not follow (i.e., pessimistic condition) the recommended hygiene and social distancing rules (e.g., whether they thought they were going to stay at their home). For each participant and week, we subtracted optimistic estimates from pessimistic estimates. We then averaged the output variables over the 4 weeks. The measure was log-transformed because of its right-skewed distribution, with higher values indicating higher perceived effectiveness of social distancing.

### Procedure

In a pretest study conducted 2 weeks before the main online experiment, participants completed three measures of individual differences in multiple numeric competencies: statistical numeracy (Cokely et al., 2012), approximate numeracy (Peters and Bjälkebring, 2015; Sobkow et al., 2019; Sobkow et al., 2020b), and subjective numeracy (Fagerlin et al., 2007).

During the main experiment (conducted from March 26^th^ to March 28^th^^2^), participants completed a questionnaire asking about demographics (age, sex, number of children, and employment status). Participants were then randomly assigned to one of the five experimental conditions described above, and they were asked to: (1) estimate the actual number of SARS-CoV-2 cases in Poland, (2) forecast the number of SARS-CoV-2 cases for consecutive 4 weeks, and (3) provide optimistic as well as pessimistic estimates of SARS-CoV-2 cases. Finally, participants completed other COVID-19 related measures in random order, including intentions toward preventive behaviors, emotional responses to the COVID-19 pandemic, sources of worry about the COVID-19 pandemic, panic buying, statistics stalking, controllability, and risk perception of COVID-19.

## Results

### The Relationships Among Measures Used in the Study

The relationships among measures used in the study are summarized in Table 1. We found that only approximate numeracy, but not statistical or subjective numeracy, was associated with participants’ intentions to take preventive behaviors (*r* = 0.14, *p* = 0.023). Subjective and approximate numeracy were also related to the perceived effectiveness of social distancing (*r* = 0.18, *p* = 0.004 and *r* = 0.13, *p* = 0.037, respectively)—people with higher subjective and approximate numeracy found obeying hygiene and social distancing rules more effective, which was associated with higher intentions to take preventive behaviors (*r* = 0.18, *p* = 0.004).

**TABLE 1:** Pearson’s *r* correlation coefficients among measures used in the study.

	*M* (*SD*)	1	2	3	4	5	6	7	8	9	10	11	12	13
1. Intentions toward preventive behaviors	5.92 (0.83)	–												
2. Statistical numeracy	1.19 (1.17)	–0.03	–											
3. Subjective numeracy	28.55 (9.15)	0.01	0.45***	–										
4. Approximate numeracy	3.25 (0.62)	0.14*	0.27***	0.36***	–									
5. Emotional responses to COVID-19	4.09 (1.56)	−0.31***	0.05	0.12	0.03	–								
6. Worry—health	4.72 (1.24)	0.29***	–0.11	−0.14*	–0.07	−0.62***	–							
7. Worry—restrictions	4.12 (1.28)	−0.19**	–0.06	0.01	–0.10	−0.17**	0.38***	–						
8. Worry—financial	4.85 (1.31)	0.09	–0.06	0.07	0.06	−0.22***	0.35***	0.48***	–					
9. Panic buying	0.02 (4.32)	0.12*	0.06	0.04	0.05	–0.10	0.03	0.13*	0.07	–				
10. Statistics stalking	3.85 (1.13)	0.17**	–0.10	–0.10	0.05	−0.29***	0.33***	0.01	0.11	0.02	–			
11. Controllability	5.55 (0.98)	0.35***	0.01	0.02	0.10	–0.08	0.04	−0.14*	–0.01	0.07	0.21***	–		
12. Risk perception	3.68 (0.72)	0.40***	–0.06	–0.05	0.01	−0.62***	0.64***	0.12	0.26***	0.12	0.34***	0.14*	–	
13. Perceived effectiveness of social distancing	7.91 (2.09)	0.18**	0.08	0.18**	0.13*	–0.11	0.07	–0.12	–0.04	0.04	0.23***	0.22***	0.10	–
14. COVID-19 forecasts	16.49 (1.78)	0.06	0.00	0.08	–0.02	0.06	–0.03	0.06	–0.05	–0.02	−0.21**	–0.03	–0.09	0.11

*p < 0.05, **p < 0.01, ***p < 0.001.

In general, intentions toward preventive behaviors were related to measures of emotional responses to COVID-19, but people were also more likely to take preventive measures when they perceived risk as higher (*r* = 0.40, *p* < 0.001), reported that they have more control over the current pandemic situation (*r* = 0.35, *p* < 0.001), and consulted with COVID-19 statistics more often (*r* = 0.17, *p* = 0.007). Furthermore, participants who declared that they inspected statistics about COVID-19 more often (scored higher on the statistics stalking measure), were also more worried about their health (*r* = 0.33, *p* < 0.001), expressed more negative emotional responses to COVID-19 (*r* = −0.29, *p* < 0.001), and perceived risk as higher (*r* = 0.34, *p* < 0.001). Interestingly, such people felt more control over the current situation (*r* = 0.21, *p* < 0.001), perceived effects of social distancing as more meaningful (*r* = 0.23, *p* < 0.001), and provided more accurate forecasts of SARS-CoV-2 cases in Poland (*r* = −0.21, *p* < 0.001).

### Factors Predicting Intentions Toward Preventive Behaviors

To predict intentions toward preventive behaviors, we ran a hierarchical regression analysis (Table 2). In the first step, we introduced the three measures of multiple numeric competencies (*R*^2^ = 0.03). We found that approximate numeracy was the only significant predictor of intentions toward preventive behaviors (*b* = 0.23, *p* = 0.014). People who were more precise in mapping symbolic numbers onto a number line were more willing to take preventive measures against COVID-19. In the second step, we introduced the experimental conditions as dummy-coded variables with the control condition as a reference (*R*^2^ = 0.05). We found that participants who were instructed to imagine the positive consequences of COVID-19 outbreak, were less willing to take preventive measures (*b* = −0.38, *p* = 0.021). None of the other conditions influenced intentions toward preventive behaviors^3^.

**TABLE 2:** Linear regression models predicting intentions toward preventive behaviors.

		Model 1					Model 2					Model 3				
Step	Coefficient	*b*	*SE*	b*	*t*	*p*	*b*	*SE*	b*	*t*	*p*	*b*	*SE*	b*	*t*	*p*
	Intercept	6.77	0.40		16.97	<0.001	6.91	0.41		17.06	<0.001	3.57	0.76		4.71	<0.001
1: Numeracy	Statistical numeracy	–0.04	0.05	–0.06	–0.87	0.384	–0.05	0.05	–0.06	–0.90	0.368	–0.04	0.04	–0.05	–0.83	0.409
	Subjective numeracy	0.00	0.01	–0.02	–0.32	0.753	0.00	0.01	–0.03	–0.41	0.681	0.00	0.01	0.01	0.19	0.849
	**Approximate numeracy**	**0.23**	**0**.**09**	**0.17**	**2.47**	**0**.**014**	**0.23**	**0**.**09**	**0.17**	**2.53**	**0**.**012**	0.12	0.08	0.09	1.57	0.119
2: Interventions	Self-efficacy condition						–0.11	0.16	–0.05	–0.69	0.488	–0.04	0.14	–0.02	–0.26	0.795
	**Positive mental imagery condition**						**−0.38**	**0**.**16**	**−0.18**	**−2.32**	**0**.**021**	–0.24	0.14	–0.12	–1.72	0.087
	Visual aid (Poland)						–0.01	0.16	0.00	–0.04	0.970	–0.12	0.14	–0.06	–0.85	0.398
	Visual aid (several countries)						–0.06	0.16	–0.03	–0.39	0.700	–0.02	0.14	–0.01	–0.17	0.868
3: Responses to COVID-19	Emotional responses to COVID-19											–0.04	0.04	–0.07	–0.95	0.344
	**Worry—health**											**0.15**	**0**.**06**	**0.22**	**2.60**	**0**.**010**
	**Worry—restrictions**											**−0.21**	**0**.**04**	**−0.32**	**−4.77**	**<0.001**
	Worry—financial											0.06	0.04	0.10	1.52	0.130
	Panic buying											0.02	0.01	0.10	1.82	0.070
	Statistics stalking											–0.02	0.05	–0.03	–0.42	0.675
	**Controllability**											**0.22**	**0**.**05**	**0.26**	**4.65**	**<0.001**
	**Risk perception**											**0.21**	**0**.**09**	**0.18**	**2.38**	**0**.**018**
	Perceived effectiveness of social distancing											0.01	0.02	0.03	0.49	0.624
	**COVID-19 forecasts**											**0.05**	**0**.**03**	**0.11**	**2.02**	**0**.**045**
	*R*^2^	0.03					0.05					0.37				

b, unstandardized beta coefficient; b*, standardized beta coefficient. Significant predictors are in bold font.

In the last step of the analysis, we introduced all measures regarding psychological responses to COVID-19, which significantly increased the model fit (*R*^2^ = 0.37). Firstly, we found that the two components of worry significantly predicted intentions toward preventive behaviors. Importantly, participants who were more worried about their health were also more willing to obey strict hygiene and social distancing restrictions (*b* = 0.15, *p* = 0.010). This relationship was reversed in the case of worry about restrictions. That is, participants who were more worried about the possible effects of restrictions introduced by the government, reported a lower willingness to take preventive measures (*b* = −0.21, *p* < 0.001). Secondly, the results indicated that higher perceived controllability of COVID-19 threat and a higher perceived risk were related to intentions toward preventive behaviors. Participants who declared that their perceived controllability of the pandemic is higher (*b* = 0.21, *p* < 0.001) and rated perceived risk as higher (*b* = 0.22, *p* = 0.018), were also more willing to take preventive measures.

Last but not least, we found that the accuracy of COVID-19 forecasts predicted the willingness to take preventive measures. In particular, participants whose individual estimates of COVID-19 spread in Poland deviated more from the actual dynamics of the pandemic (i.e., people who were less accurate in forecasting the increase of SARS-CoV-2 cases in Poland) were also more likely to take preventive measures (*b* = 0.05, *p* = 0.045). The pattern of results held when we adjusted the model for demographic measures such as age and gender. Willingness to take preventive measures increased with age, *b* = 0.01, *p* = 0.014, and females were more willing to take preventive measures, *b* = −0.26, *p* = 0.064.

## Discussion

In the current study, we investigated which factors may be related to behavioral intentions toward COVID-19 preventive behaviors among young adults. Four main conclusions can be drawn from the results. First, we observed very weak or insignificant relationships between numeracy and measures associated with the COVID-19 outbreak. Second, none of our experimental manipulations revealed the potential to be applied in order to increase behavioral intentions among young adults. The only significant relationship we found in this context suggested that positive mental imagery may decrease preventive behaviors. Third, preventive behaviors were best predicted by a combination of different types of worry, controllability, and risk perception. Individuals who were worried about health, perceived risk as higher but also believed they could mitigate this risk, were more prone to obey strict hygiene and social distancing rules for a longer time (e.g., 3 months). Importantly, worry about the restrictions was negatively related to behavioral intentions. Finally, we found quite surprising but very intriguing results regarding a new measure—statistics stalking. On the one hand, individuals who searched for new statistics more often were more worried about their health and assessed the risk as higher. On the other hand, they were also more accurate in their COVID-19 forecasts, perceived effectiveness of social distancing as higher, and had higher protective behavioral intentions.

### Insignificant or Weak Relationships With Numeracy and the Experimental Manipulations

In other studies concerning diverse health contexts, numeracy (in its different components) has consistently been related to risk perception, affective responses to risks, more accurate understanding of risks, and better (evidence-based) decisions (Reyna et al., 2009; Garcia-Retamero et al., 2019). However, in the current research, the different numerical competencies showed small or insignificant correlations with intentions toward preventive behaviors and the other variables related to the pandemic response. In particular, approximate numeracy was the only competency significantly predicting intentions (albeit not in the final regression model). In our previous research, we have found that, among the different numerical competencies, approximate numeracy was the strongest predictor of perceived risks and affective reactions (Petrova et al., 2019) and superior decision making beyond fluid intelligence (Sobkow et al., 2020b); it also successfully improved performance on some daily math-related tasks following a brief training (Sobkow et al., 2019). However, in the context of intentions toward the COVID-19 preventive behaviors, it was not among the most important variables.

Similar null (or puzzling) findings were obtained with regard to the three types of interventions that had been previously effective in a number of other contexts: mental imagery (Sobkow et al., 2016), self-efficacy (Sheeran et al., 2016), and visual aids (Garcia-Retamero and Cokely, 2017).

Interestingly, we found that people may become less willing to engage in preventive behaviors if they produce positive mental imagery about the future (however, this effect was not significant in the final model containing other COVID-19-related measures). This finding is consistent with the results of other studies showing that positive mental imagery is related to more optimistic forecasts and “rose-colored” risk perceptions. For example, Neck and Manz (1992, 1996) showed that more positive images of the future led to more positive emotional experiences in entrepreneurs and motivated them to think about business in terms of opportunities rather than obstacles. It is possible that people who are more prone to imagine positive consequences of the pandemic (e.g., easily produce images related to spending more time at home with a family) tend to undervalue risk associated with the health threat and, as a result, are less willing to undertake preventive actions. Future studies should also investigate the potential role of negative mental images, which might be expected to produce opposite effects on both risk perception and protective behaviors.

It is worth noticing that we did not include manipulation checks (e.g., ratings of the subjective vividness of mental images generated by participants or their understanding of visual aids). So there could be design and implementation issues with interventions that could offer potential explanations for their limited effectiveness. Nevertheless, without these control measures, we are unable to identify them accurately.

The general pattern of null findings from the interventions and numerical competencies also suggests that the context of a pandemic, characterized by extreme information overload, uncertainty, and worry, could create a decision environment in which factors traditionally found to influence preventive intentions are “trumped” by other specific contextual influences (e.g., see also Erceg et al., 2020; Thompson et al., 2020 non-peer-review preprints). For instance, in the current study, worry (in its different forms) was a strong predictor of intentions.

### Worry and Risk Perception

The fact that worry and risk perception were found to be main predictors of behavioral intentions is not surprising in the light of previous research and theories (Loewenstein et al., 2001; Slovic et al., 2007; Bruine De Bruin and Bennett, 2020; Zaleskiewicz and Traczyk, 2020). Additionally, a recent study by Thompson et al. (2020; a non-peer-reviewed preprint) showed a positive link between worry and COVID-19 risk perception, while Erceg et al. (2020; a non-peer-reviewed preprint) revealed that worry is an important predictor of more responsible behaviors in case of the pandemic. Nevertheless, we argue that our results shed new light on the role of worry in risk perception and in promoting preventive behaviors. In Erceg et al. (2020) study, the worry was measured using a single item (“Considering all the known aspects of the current situation, how worried would you say you are for yourself and your family because of the coronavirus?”), while we found that “worry because of coronavirus” is not unitary. We observed three types of worry that, even if positively correlated, were differently linked with COVID-19 related measures. The first type of worry—worry about health—is probably the most prototypical in case of the pandemic (e.g., Thompson et al., 2020 asked about the extent of worry that oneself and close others would be infected with COVID-19). In our study, individuals who were more worried about health (theirs or their family members), assessed risk related to coronavirus as higher, checked statistics more often, and had higher behavioral intentions toward preventive behaviors.

On the other hand, the second type of worry—worry about restrictions and personal freedom (e.g., being unable to meet with friends)—was positively related to panic buying and negatively to controllability, but importantly, also negatively linked to behavioral intentions. This type of worry seems to be of particular importance among young adults and adolescents to whom interaction with peers is especially important (Andrews et al., 2020). Because of that, adherence to social distancing rules may be particularly challenging for them. It would be worth to develop interventions and appeals targeting young people and their worries. For example, Van Bavel et al. (2020) suggested that we should use the term “physical distancing” instead of “social distancing” because the latter implies that one should cut off all interactions. “Physical distancing” is preferred because it stresses physical separation. However, social connections are still possible (e.g., using social media or other technology allowing temporally synchronous and informationally rich connection using the internet). Finally, the third type of worry—the worry about finance—was only related to higher risk perception but not to other COVID-19 related measures. The idea that worry is not unitary reflects the factor structure found in the Worry Domains Questionnaire (McCarthy-Larzelere et al., 2001). Moreover, three types of worry related to the pandemic found in our study seem to be closely related to three domains that were identified among others from the abovementioned questionnaire: physical threat, relationships, and financial.

The above-reviewed results provoke questions about practical implications: should we try to increase worry to achieve higher compliance with the protective measures (as a lesser of two evils)? We argue that during the pandemic, people already experience an elevated level of distress and chronic anxiety, especially when they are put on quarantine (Brooks et al., 2020; Van Bavel et al., 2020). Moreover, when people are faced with a real and serious threat that has the capacity to evoke strong fear (such as the one related to the COVID-19 pandemic; Van Bavel et al., 2020), they may not react to information about the size of the threat, even if it is presented to them in a relatively “friendly” and easy-to-understand format (such as our visual aids). Prior research supports such reasoning. It has indicated that those individuals who experience excessively high health anxiety, demonstrate various non-rational behaviors, i.e., they may avoid consulting with a physician because they regard clinics as a source of contagion and sickness rather than a place providing help (Lee, 2014; Taylor, 2019). Moreover, people with high health anxiety are often alarmed by uninformative signals which can make them overestimate the seriousness of potential illness (Wheaton et al., 2010; Hedman et al., 2016) and tend to misinterpret health-related stimuli (Taylor and Asmundson, 2004; Wheaton et al., 2012). Therefore, we hypothesize that in situations in which people are exposed to severe threats, risk communication should be preceded by actions oriented on lowering the anxiety level. However, future research should test this issue empirically.

It is worth noting that our study was conducted at the beginning of the outbreak in Poland, when the level of worry was particularly elevated. Now (July, 2020), most of the countries after weeks of lockdown face the next challenge—how to make a safe transition to the “new normal” (Habersaat et al., 2020). Until a vaccine or effective treatment becomes available, societies must still use special hygiene as well as social and physical distancing measures to control the spread of the virus. Nevertheless, these protective behaviors are associated with high social and economic costs. Recently, a group of experts from diverse academic disciplines (Habersaat et al., 2020) proposed 10 recommendations to manage COVID-19 transition. One of these important considerations was to increase resiliency and self-efficacy.

### Controllability and Self-Efficacy

In our study, we also attempted to investigate the relationships among different measures related to controllability and self-efficacy. We found that self-reported controllability of the pandemic was associated with higher perceived effectiveness of social distancing as well as with higher intentions toward protective behaviors. These results, while encouraging, should be taken with some caution because the measure of controllability used in this study had relatively low reliability. Moreover, our (relatively simple) experimental manipulation of self-efficacy was found to be ineffective. Future research should address these problems in a more detailed manner. For example, Habersaat et al. (2020) argued that we should distinguish self-efficacy (the belief that an action can be completed) and response efficacy (the belief that action can reduce a threat). One could design more powerful interventions in which participants would be educated what and why it should be done to increase self-efficacy and response efficacy (Habersaat et al., 2020). Moreover, in future interventions, it would be worth focusing on various psychological mechanisms such as self-monitoring, feedback on performance, contingent rewards, prompting of behavioral goals, and planning social support. Similar actions were found to be effective in building self-efficacy in dietary (Prestwich et al., 2014) or physical activity (Ashford et al., 2010) interventions. Moreover, these interventions should be reinstated in case of future waves of infection (Habersaat et al., 2020).

### Statistics Stalking

Last but not least, we observed in this study interesting effects related to a new measure—statistics stalking. The idea of statistics stalking was introduced by Ellen Peters in a *New York Times* article entitled “Is Obsessing Over Daily Coronavirus Statistics Counterproductive?” (Peters, 2020). Peters argued, based on a survey conducted at the beginning of the outbreak in the United States, that statistics stalkers—individuals who checked coronavirus data every day—were more anxious and assessed the chances of being infected with the SARS-CoV-2 as higher. Moreover, they were more prone to amassing supplies (such as water or toilet paper) and buying surgical masks, which could be seen as overprotection. In our study, we observed similar results: participants who checked coronavirus statistics more often, experienced more negative emotions, were more worried about the health, and assessed risk related to the COVID-19 pandemic as higher.

Nevertheless, we also found that statistics stalking may be related to positive measures. Individuals who searched for statistics more often felt more control over the situation, perceived social distancing as more effective, and had higher behavioral intentions toward preventive behaviors. Finally, they were also more accurate in their COVID-19 forecasts. Interestingly, statistics stalking was the only measure associated with the accuracy of predictions. The question arises, what psychological mechanisms may underlie these effects. Surprisingly, even though previous research has demonstrated that individuals with higher numeracy searched for more information in the decision-from-experience paradigm (Ashby, 2017; Traczyk et al., 2018a), none of the numeracy measures used in our study was related to searching for new statistics about the coronavirus. Future studies are needed to delve into this topic. However, a recent study by Atanasov et al. (2020) gave substantial suggestions regarding cognitive mechanisms that may underlie better forecasts. In their study, most accurate real-life forecasters made frequent and small revisions allowing them to build a better understanding of uncertain situations. Probably, individuals who searched for new statistics more often also learned the structure of the environment. This process could be deliberative (e.g., in Atanasov et al., 2020, high-frequency updaters scored higher on the crystallized intelligence measure). But there is also evidence that people could non-intentionally learn complex patterns (Reber, 1993; Sobkow et al., 2018), covariances (Catena et al., 1998), and probabilities (Traczyk et al., 2019).

### Limitations

First, the primary outcome variable in this study tapped intentions toward preventive behavior and not real behavior. Meta-analyses (Sheeran, 2002; Webb and Sheeran, 2006; Sheeran and Webb, 2016) showed that while intentions and behavior are usually moderately correlated, there is a gap between these two constructs (people do not always do what they declared they would do). However, we argue that the role of behavioral intentions is nontrivial—they significantly mediate the effect of interventions (e.g., changing attitudes, social norms, and self-efficacy) on behavior (Webb and Sheeran, 2006; Sheeran et al., 2016)—and thus could be used a proxy of preventive behaviors. We also argue that a measure of actual preventive behavior could be insensitive during the lockdown—because people were forced to stay at home, pronounced ceiling effects would be observed. Instead, we asked people for their intentions to keep these behaviors in the long-term to increase the sensitivity of the measure.

Second, because our study was conducted under extraordinary conditions (the beginning of the pandemic in Poland), no validated measures of perception of this threat were available. All of the COVID-19-related measures used in this study were newly developed and not tested in previous research. Nevertheless, most of them (except for controllability) had satisfactory reliability and the structure of correlations among them suggests their validity.

Third, the number of cases/deaths has been changing dynamically, depending on restrictions announced by the authorities as well as people’s behavior. When we collected data, the number of SARS-CoV-2 cases in Poland had been changing linearly (approximately 350 new cases/day), and there was a plateau for a few months. Nevertheless, from July/August, the beginning of the “second wave” of the outbreak could be observed. People’s psychological reactions to this threat (e.g., worry, risk perception, behavioral intentions) have been changing throughout the pandemic, suggesting that this problem (the difficulty in studying people’s psychological reactions to the pandemic) yields to longitudinal research [such as COSMO project in Germany (COVID-19 Snapshot Monitoring); Betsch et al., 2020].

Fourth, this study was conducted on a specific sample (relatively young Polish college students) but not a representative one, so one should be careful in generalizing the results of this study. Finally, although we used the risk-as-feelings hypothesis (Loewenstein et al., 2001) as our main theoretical framework, this study was largely exploratory and was not aimed to confirm a particular prediction of this model. Nevertheless, we believe that including a broad set of various measures in the study is promising in the exploration of possible factors that influence protective behaviors during the pandemic and may help cope with this novel and severe threat in future.

### Summary

Previous research suggests that it is crucial to identify which factors can motivate young adults to comply with the recommended preventive measures against the coronavirus pandemic. Even though they may be less likely to suffer health consequences from the virus as a group, they can still transmit it to more vulnerable individuals. Our study sheds new light on this issue by suggesting different sources of worry related to the COVID-19 outbreak (i.e., health, restrictions, and financial) in predicting willingness to take preventive measures in this population. Besides this important theoretical notion, our results have the potential to be applied to the design of novel and effective interventions and policies, for example, by decreasing people’s susceptibility to create excessively positive mental imagery of the situation because highly optimistic mental images may hamper the willingness to undertake protective behaviors.

## Data Availability Statement

The datasets generated for this study can be found in the online repositories. The names of the repository/repositories and accession number(s) can be found below: https://osf.io/ef3na/.

## Ethics Statement

The studies involving human participants were reviewed and approved by the Ethics Committee at Faculty of Psychology in Wrocław, SWPS University. The patients/participants provided their written informed consent to participate in this study.

## Author Contributions

AS, JT, TZ, RG-R, and DP developed the study concept and contributed equally to the study design. AS and JT conducted the study and collected data. JT performed the data analysis. AS, JT, TZ, RG-R, and DP interpreted the results, wrote, and reviewed the article. All authors approved the final version of the manuscript.

## Conflict of Interest

The authors declare that the research was conducted in the absence of any commercial or financial relationships that could be construed as a potential conflict of interest.

## Footnotes

1 https://github.com/CSSEGISandData/COVID-19

2 The main study was conducted 3 weeks after the first SARS-CoV-2 case was announced in Poland (March 4th). Authorities decided to close all schools, kindergartens, universities on March 10th and more strict “lockdown” type restrictions such as prohibiting non-essential travels (except traveling to work or home) or public gatherings larger than two people (except families) were announced on March 24th (2 days before data collection started).

3 In a series of ANOVA, we tested whether our experimental conditions impact other measures used in the study. There were no significant effects of experimental condition on any other measure.
